# A systematic review of randomized controlled trials of the Wenyang Huoxue method in treating diabetic peripheral neuropathy

**DOI:** 10.1097/MD.0000000000017618

**Published:** 2019-10-18

**Authors:** Zhiyuan Deng, Manjia Wang, Yao-hua Fan, Ruilin Huang, Yawen Rao, Xurong Mai, Min Liu

**Affiliations:** a Guangzhou University of Chinese Medicine; b The First Affiliated Hospital of Guangzhou University of Chinese Medicine, Guangzhou, China.

**Keywords:** diabetic peripheral neuropathy, systematic analysis, Wenyang Huoxue method

## Abstract

**Objective:**

To assess the efficacy and safety of the Wenyang Huoxue method for patients with diabetic peripheral neuropathy.

**Methods:**

A systematic literature search was performed using 7 databases: PUBMED, EMBASE, the Chinese National Knowledge Infrastructure, Wanfang, Chinese BioMedical, and the VIP Chinese Science and Technique Journals. The publication time was from the start of each database up to November 2018. Review Manager 5.3 software was used for assessing potential bias, data synthesis, and the subgroup analysis. Begg and Egger tests were used to assess funnel plot symmetries using Stata 14.0 software. The Grading of Recommendations Assessment, Development and Evaluation (GRADE) system was used to assess the quality of evidence.

**Results:**

A total of 22 trials involving 1835 participants were eligible. There were significant differences in a total effective rate between the Wenyang Huoxue method combined with Western medicine and Western medicine alone (RR = 1.33, 95% CI 1.26–1.41; *P* < .00001). As for the sensory conduction velocity (SCV) of the peroneal nerve, the Wenyang Huoxue method combined with Western medicine compared with Western medicine alone had a significant increase (weighted mean difference [WMD] = 5.00, 95% CI 3.42–6.57; *P* < .00001). Also, the Wenyang Huoxue method combined with Western medicine had significant increases in motor conduction velocity (MCV) of the peroneal nerve (WMD = 4.48, 95% CI 3.78–5.19; *P* < .00001), tibial nerve SCV (WMD = 3.47, 95% CI 2.66–4.28; *P* < .00001), tibial nerve MCV (4.87, 95% CI 3.21–6.53; *P* < .00001), median nerve SCV (WMD = 3.78, 95% CI 3.07–4.50; *P* < .00001), and median nerve MCV (WMD = 4.50, 95% CI 3.40–5.59; *P* < .00001). However, the effect of the Wenyang Huoxue method on fasting blood glucose, 2-h postprandial blood glucose, and glycosylated hemoglobin was not statistically significant. Egger's test results showed that there was no publication bias (*P* = .0008), but the trim and filling method showed steady results. An influence analysis showed that no single study affected the overall result. The GRADE quality of the evidence was low to moderate across the different outcomes.

**Conclusion:**

Despite of the apparently positive findings, the quality of GRADE is not high, suggesting that the Wenyang Huoxue method can improve nerve conduction velocity to a certain extent, but more rigorous literature is needed to support this evidence.

## 1 Introduction

Diabetes mellitus (DM) has a very high incidence worldwide. The World Diabetes Federation reported that there were about 451 million diabetic patients worldwide in 2017; its prevalence is expected to soar to 693 million in 2045.^[1]^ DM is accompanied by multiple complications, of which diabetic peripheral neuropathy (DPN) is the most common. Between 10% and 20% of patients with DM are also diagnosed with DPN.^[2]^ The main clinical features of DPN include limb sensation and neurological dysfunction, manifesting as limb dissonance, numbness, muscle weakness, urgency pain, decreased tendon reflex, muscle atrophy, and spasms. It increases the disability rate of diabetic patients and greatly reduces their quality of life.^[3]^

DPN incidence is related to issues such as hyperglycemia, microvascular disease, lipid metabolism disorder, oxidative stress damage, and blood–nerve barrier factors; thus, it has characteristics of repeated prolongation.^[4,5]^ Notably, mecobalamin, lipoic acid, epalrestat, physical therapy, and stem cell transplantation have a certain effect, but have not yet achieved satisfactory results.^[6]^ Alternatively, traditional Chinese medicine (TCM) encompasses a holistic treatment and has many active ingredients; hence, it can interfere with the occurrence and development of diseases through the combination of single target and multi-target synergy. Therefore, the effectiveness and safety of treating DPN with TCM has been increasingly valued by the medical community.

According to the clinical symptoms of DPN, it can be classified into the “Bi Syndrome” and “Blood Bi” categories of TCM. The pathogenesis of DPN involves a deficiency of Yang Qi, cold coagulation, and blood stasis. Therefore, the Wenyang Huoxue method is an important measure to treat DPN.

Many clinical trials^[7,8]^ in China have proven that the Wenyang Huoxue method can treat DPN effectively and safely; however, due to the small sample size, there is not enough evidence to support its routine application extensively. In addition, a systematic review of the efficacy and safety of the Wenyang Huoxue method in DPN treatment has not been reported. Thus, this systematic review and meta-analysis of clinical trials will evaluate the evidence on the efficacy, safety, and potential advantages of the Wenyang Huoxue method for treating DPN.

## 2 Method

### 2.1 Types of research

The Wenyang Huoxue method for treating DPN was not limited to a particular type of study regarding a single- or double-blind approach; only Chinese and English literature was reviewed. However, animal experiments and reviews were excluded.

### 2.2 Research criteria

Diagnostic criteria included a diagnosis of DPN according to the “Diabetes Clinical Practical Guidelines.”^[9]^ Exclusion criteria consisted of diabetic ketoacidosis, hyperosmolar coma, stress, infection, hemoglobin abnormalities, and other diseases affecting glucose metabolism; severe diabetic complications, stroke, mental illness, and other diseases caused by peripheral neuropathy such as cervical spondylosis as well as drugs or other occupational factors were also considered criteria for exclusion. Furthermore, pregnant or lactating women, drug allergy patients, or patients with tumors, severe liver kidney dysfunction, or other diseases were excluded. Since this meta-analysis is based on data from previously published studies, ethical approval and patient consent were not required.

### 2.3 Types of intervention

The control group comprised patients given conventional treatments such as lipoic acid, alprostadil, vitamins, insulin, exercise therapy, and diet therapy. Patients in the treatment group were treated with the Wenyang Huoxue method and Western medicine. The drug regimen in the treatment group should be consistent with the control group. There were no other treatments in the treatment group, and tests including other Chinese medicine interventions (such as Chinese medicine decoction and acupuncture) were excluded.

### 2.4 Types of measurement results

The main test results included total effectiveness, sensory conduction velocity (SCV) and motor conduction velocity (MCV) of the peroneal nerve, SCV and MCV of the tibial nerve, SCV and MCV of the median nerve, fasting blood glucose (FBG), 2-h postprandial blood glucose, and glycosylated hemoglobin.

### 2.5 Search strategy

#### 2.5.1 Electronic searches

The search databases included PUBMED, EMBASE, the Chinese National Knowledge Infrastructure, Wanfang, Chinese BioMedical, and the VIP Chinese Science and Technique Journals. The publication time was from the start of each database up to November 2018. The Chinese search terms and keywords “Wenyang Huoxue,” “warming yang,” or “activating blood circulation” and “diabetic peripheral neuropathy,” “peripheral neuropathy,” or “diabetes mellitus” were used. According to the characteristics of different databases, all randomized controlled trials (RCTs) of the Wenyang Huoxue method combined with Western medicine in the treatment of DPN were retrieved by combining the corresponding advanced retrieval and free word retrieval methods.

#### 2.5.2 Other searches

We also searched the International Clinical Trial Registration Platform, the China Clinical Trial Registration Platform, and the reference sections for each study. In addition, we retrieved a conference paper on the use of Wenyang Huoxue method for DPN.

### 2.6 Data collection and analysis

#### 2.6.1 Data collection

**Figure 1:**
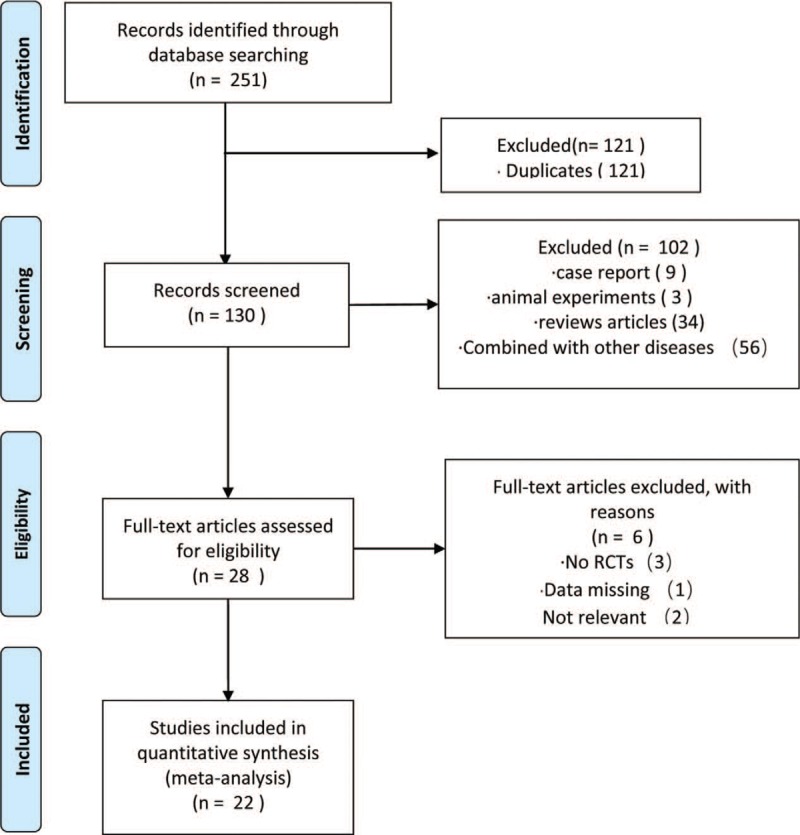
PRISMA flow chart.

Based on the research criteria and search strategies identified above, 2 reviewers reviewed the topics and abstracts separately and independently selected possible references. Two reviewers used standardized data sheets to extract data including basic information such as research, patients, interventions, and outcomes. If the reviewers encountered inconsistencies, they would be resolved in a consultation with a third reviewer. See the PRISMA flow chart for the research selection (Fig. 1).

#### 2.6.2 Assessing the risk of bias

Two reviewers used the Cochrane Handbook version 5.2 to assess the risk of bias in all included studies by considering random sequence generation, allocation concealment, blindness to participants, personnel, outcome evaluators, incomplete outcome data, selective reporting, and any other sources of bias.

#### 2.6.3 Data collation and analysis

We used the statistical software RevMan 5.3 and Stata 14.0 from the Cochrane Collaboration. For dichotomous data, we used the risk ratios (RRs) to analyze the statistics; for continuous data, we used the weighted mean difference (WMD). All analyses involved the calculation of 95% confidence intervals (95% CIs). A heterogeneity test was performed on the included studies using the χ^2^ test. If there was no statistical heterogeneity between the studies (*P* > .05, *I*^2^ < 50%), the fixed effect model was used for meta-analysis. Otherwise, (*P* ≤ .05, *I*^2^ ≥ 50%), a random effects model was used for meta-analysis. A “funnel chart” analysis was performed to show the distribution status of the research data and to determine whether the study was published with bias.

#### 2.6.4 Subgroup analysis

Heterogeneity sources were analyzed by a subgroup analysis based on the study design and different treatment options.

#### 2.6.5 Publication bias

The Begg and Egger tests were performed using Stata 14.0 software and bias was determined to be statistically significant at *P* < .05.

#### 2.6.6 Quality assessment of evidence

We used the Grading of Recommendations Assessment, Development and Evaluation (GRADE) scoring system to assess the quality of evidence.^[10]^

## 3 Results

### 3.1 Research ideas

A total of 251 records were found by the electronic database search; of these, 121 were duplicates; thus, 130 were left after deleting the duplicate data. After screening the headlines and abstracts, a total of 102 studies were excluded: 56 were combined with other diseases, 9 were case reports, 3 were animal experiments, and 34 were review articles. Twenty-eight articles potentially met the inclusion requirements. Since 3 cases were not RCTs, 2 cases were irrelevant, and 1 case was combined with other TCM intervention methods, only 22 studies^[8,11–31]^ met the inclusion criteria. The papers meeting the inclusion criteria had extractable data for evaluating the efficacy of the Wenyang Huoxue method combined with Western medicine in treating patients with DPN.

### 3.2 Research characteristics

The 22 included studies reported on 1835 patients with DPN, aged 30 to 72 years. All trials were published in China from 2003 to 2017; no multicenter trials were reported. See Table 1 for details.

**Table 1:**
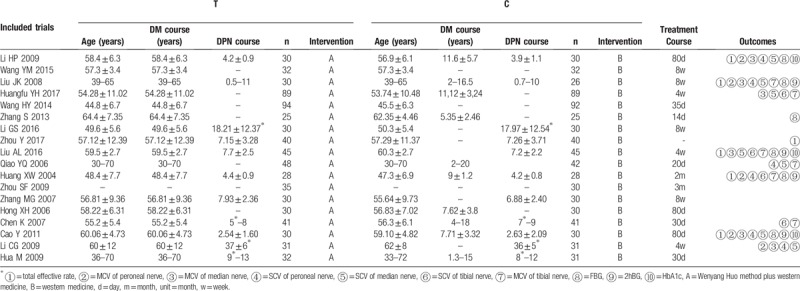
Basic characteristics of the included test.

Most trials included limited samples ranging from 50 to 100 participants. The average course of treatment in these studies ranged from 4 weeks to 80 days. Most trials (68.18%) were short-term treatments, for <3 months, while others were >3 months. One study reported follow-up. Twenty-one studies evaluated the total effective rate, 5 evaluated peroneal nerve MCV, 6 measured peroneal nerve SCV, 4 measured tibial nerve SCV, 6 measured tibial nerve MCV, 7 measured median nerve SCV, 6 measured median nerve MCV, 6 measured FBG, 4 measured 2-h postprandial blood glucose (2hBG), and 4 measured glycosylated hemoglobin (HbA1c).

### 3.3 Bias risk

**Figure 2:**
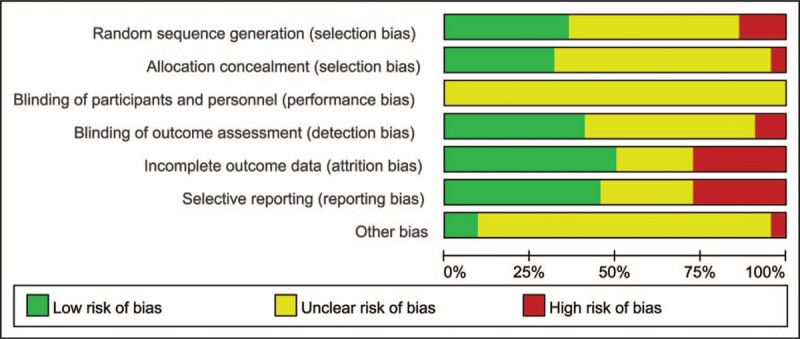
Risk of bias graph.

**Figure 3:**
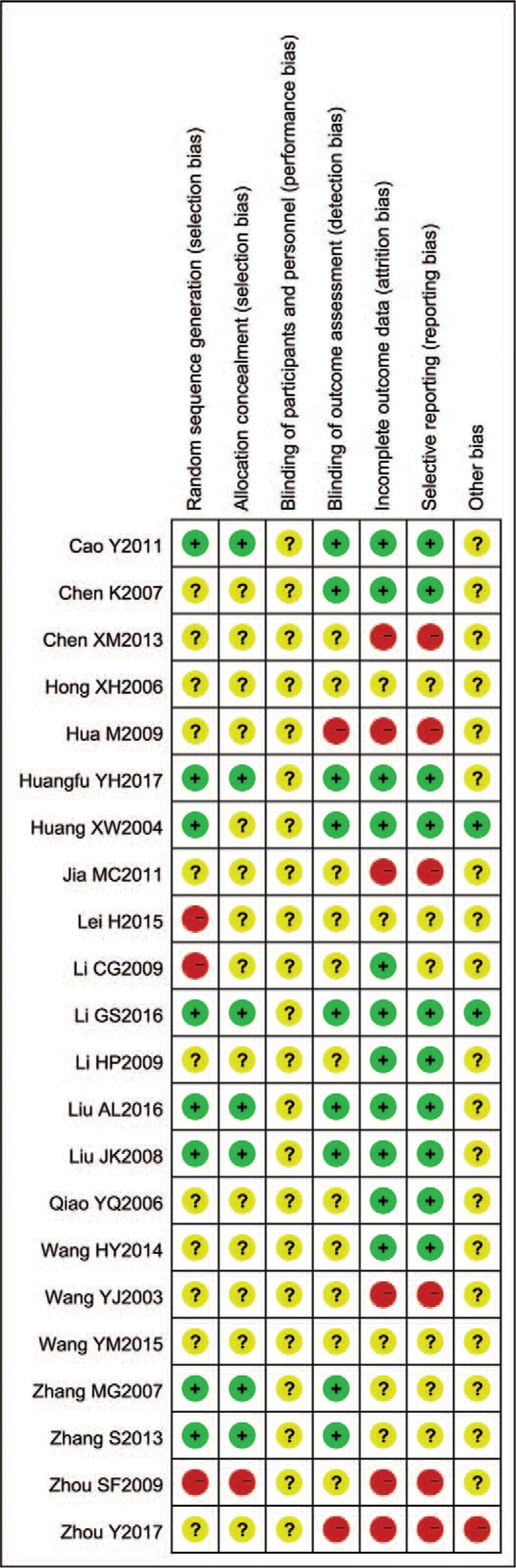
Risk of bias summary.

According to the quality evaluation criteria, each included study was evaluated and integrated into a methodological quality diagram (Figs. 2 and 3). Only 8 papers mentioned a random number table method; the rest of the trials were not detailed. One paper mentioned a single-blind experiment, but other trials did not describe the blinding details. Hence, there was a risk of ambiguity or high bias.

### 3.4 Intervention impact

#### 3.4.1 Total effective rate

**Figure 4:**
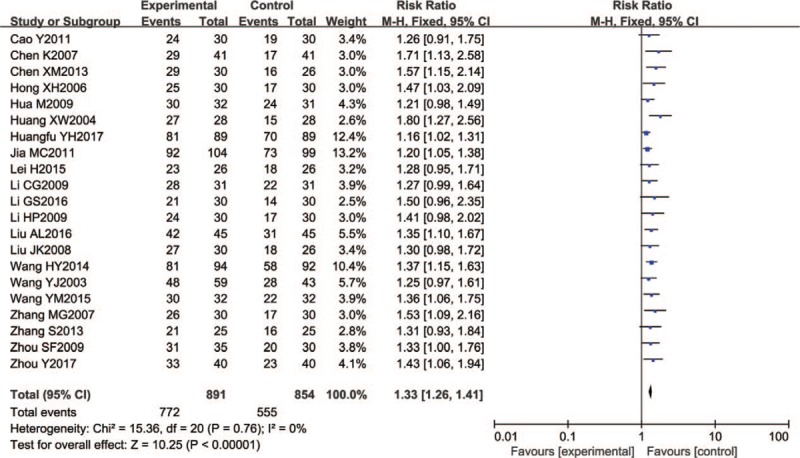
Forest chart of total effective rate.

A total of 1745 patients in 21 trials reported a total effective rate. A joint analysis with a fixed effect model showed that there were significant differences between the Wenyang Huoxue method combined with Western medicine and Western medicine alone (RR = 1.33, 95% CI 1.26–1.41; *P* < .00001). See Figure 4.

#### 3.4.2 Sensory conduction velocity of the peroneal nerve

**Figure 5:**
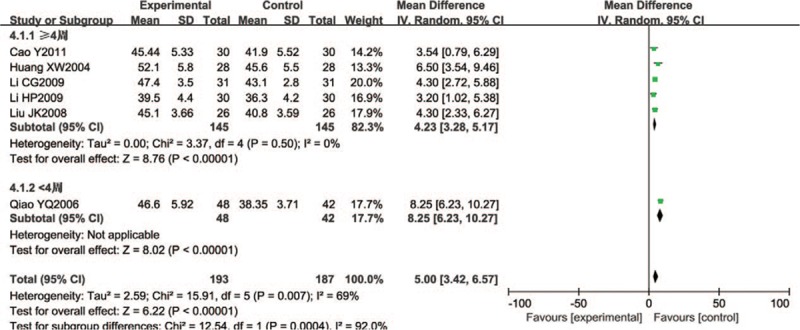
Forest chart of SCV of the peroneal nerve.

Six trials (380 participants) assessed peroneal nerve SCV. The mean difference (*I*^2^ = 69%) was summarized by a random effect model. The peroneal nerve SCV of the Wenyang Huoxue method combined with Western medicine was significantly higher than that of Western medicine alone (WMD = 5.00, 95% CI 3.42–6.57; *P* < .00001). Patients treated ≥ 4 weeks had good test results, with statistical significance (n = 290; WMD = 4.23, 95% CI 3.28–5.17; *P* < .00001), and the changes in patients treated < 4 weeks were also significant (n = 90; WMD = 8.25, 95% CI 6.23–10.27; *P* < .00001) (Fig. 5).

#### 3.4.3 Motor conduction velocity of the peroneal nerve

**Figure 6:**
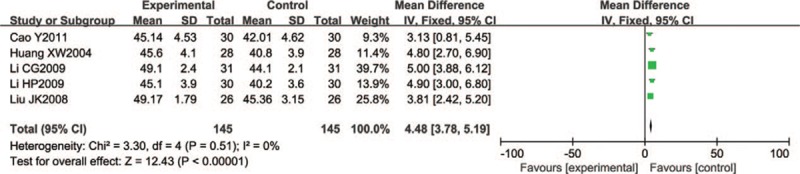
Forest chart of MCV of the peroneal nerve.

Five of the included trials (n = 290) reported peroneal nerve MCV with significant differences (WMD = 4.48, 95% CI 3.78–5.19; *P* < .00001) (Fig. 6).

#### 3.4.4 Sensory conduction velocity of the tibial nerve

**Figure 7:**
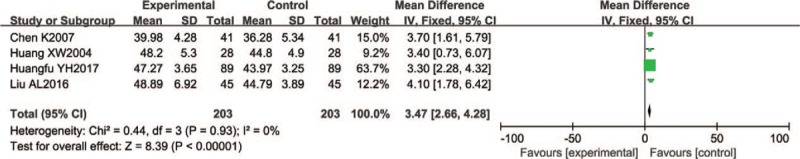
Forest chart of SCV of the tibial nerve.

Four trials (406 participants) reported tibial nerve SCV. Compared with Western medicine alone, the Wenyang Huoxue method combined with Western medicine treatment group had a statistically significant difference (fixed effect model WMD = 3.47, 95% CI 2.66–4.28; *P* < .00001) (Fig. 7).

#### 3.4.5 Motor conduction velocity of the tibial nerve

**Figure 8:**
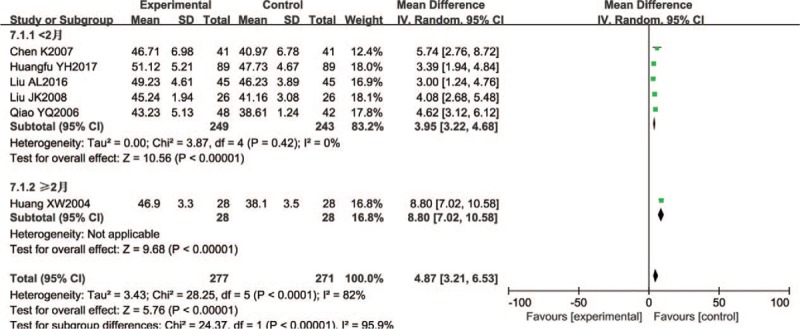
Forest chart of MCV of the tibial nerve.

A total of 548 participants in 6 studies reported tibial nerve MCV. Five trials were small sample trials, including 56 to 100 patients, and only 1 trial had a sample size greater than 100. In a joint analysis, the Wenyang Huoxue method combined with Western medicine had a better effect on tibial nerve MCV (random effect model, WMD = 4.87, 95% CI 3.21–6.53; *P* < .00001; *I*^2^ = 82%). Patients treated ≥ 2 months had good test results, with statistical significance (n = 56; WMD = 8.80, 95% CI 7.02–10.58; *P* < .00001), and the changes in those treated < 2 months were also significant (n = 492; WMD = 3.95, 95% CI 3.22–4.68; *P* < .00001) (Fig. 8).

#### 3.4.6 Sensory conduction velocity of the median nerve

**Figure 9:**
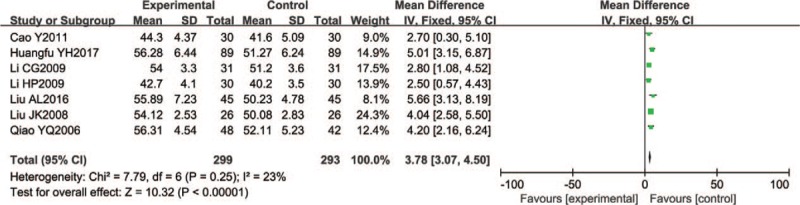
Forest chart of SCV of the median nerve.

Seven trials (592 participants) reported median nerve SCV with significant differences (fixed effect model WMD = 3.78, 95% CI 3.07–4.50; P < 0.00001; I^2^ = 23%) (Fig. 9).

#### 3.4.7 Motor conduction velocity of the median nerve

**Figure 10:**
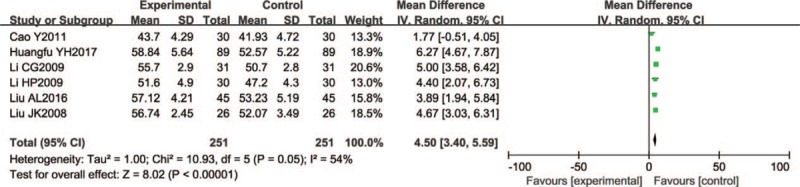
Forest chart of MCV of the median nerve.

A total of 502 participants in 6 studies reported median nerve MCV. Five trials were small sample trials, involving 50 to 100 patients. In a joint analysis, the Wenyang Huoxue method combined with Western medicine had a better effect (mean difference of random effect: 4.50, 95% CI 3.40–5.59; *P* < .00001). (*I*^2^ = 54%) (Fig. 10).

#### 3.4.8 Blood glucose index

A total of 368 participants in 6 studies reported FBG; all were small sample trials. In a combined analysis, the effect of Wenyang Huoxue method combined with Western medicine on FBG was not statistically significant (mean difference in random effect: WMD = −0.30, 95% CI −0.61 to 0.02; *P* = .06; *I*^2^ = 73%).

A total of 258 participants from 4 studies reported 2-h postprandial blood glucose; all were small sample trials. The results showed that there was no statistically significant effect of Wenyang Huoxue method combined with Western medicine on 2-h postprandial blood glucose (random effect, WMD = −0.26, 95% CI -−.84 to 0.33; *P* = .39; *I*^2^ = 65%).

A total of 312 participants in 4 studies reported glycosylated hemoglobin, 3 of which were small sample trials. The results showed that the effect of Wenyang Huoxue method combined with Western medicine on glycosylated hemoglobin was not statistically significant (random effect model, WMD = −0.51, 95% CI −1.41 to 0.39; *P* = .26; *I*^2^ = 93%).

### 3.5 Publication bias

To assess publication bias, Egger and Begg tests (Egger test *P* = .0008, Begg test P = .065) were performed; these results suggested a risk of publication bias. To further assess the risk, the trim and filling method was used to assess the studies; the results (odds ratio = 1.263, 95% CI 1.113–1.434, *P* = .000) were statistically significant, indicating that the results were steady.

### 3.6 Sensitivity analysis

**Figure 11:**
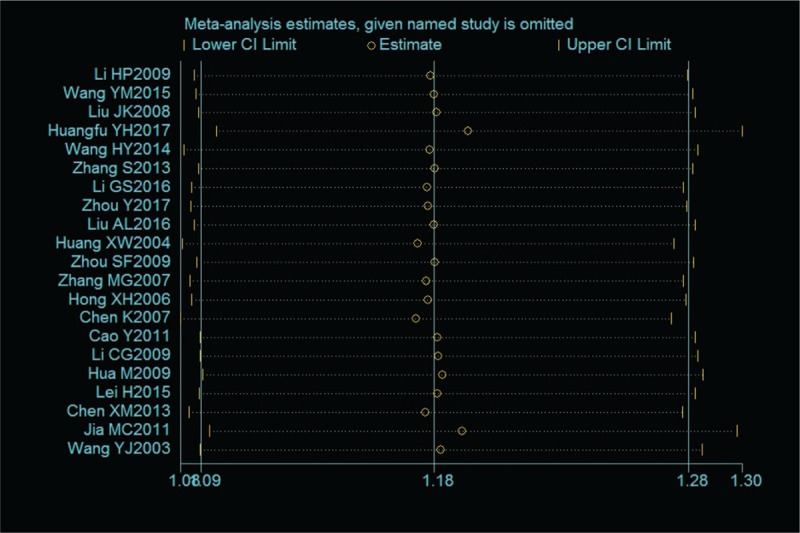
Chart of sensitivity analysis.

A sensitivity analysis was performed to assess whether a single study would affect the overall outcome. The results showed that none of the studies affected the overall outcome (Fig. 11).

### 3.7 Literature quality

We further used the GRADE Working Group's guidelines to evaluate the quality of evidence (Table 2).

**Table 2:**
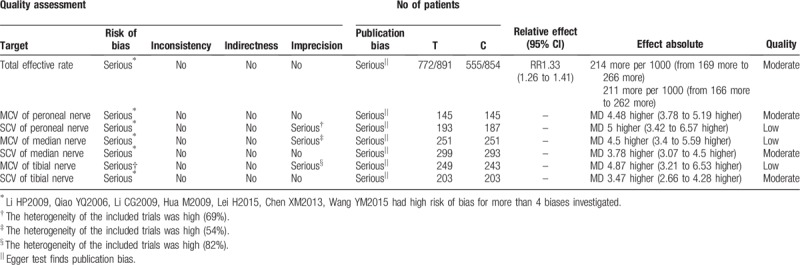
GRADE rating table.

## 4 Discussion

This meta-analysis included 22 RCT studies involving 1835 patients with DPN aged 30 to 72. The analysis revealed that the Wenyang Huoxue method combined with Western medicine can influence a total effective rate, peroneal nerve SCV and MCV, tibial nerve SCV and MCV, and median nerve SCV and MCV. However, there was no statistically significant effect on FBG (*P* = .06), 2-h postprandial blood glucose (*P* = .39), and glycosylated hemoglobin (*P* = .26). Although the reported results were positive, there was still insufficient evidence to promote the use of the Wenyang Huoxue method combined with Western medicine in patients with DPN because of the poor quality of the methods and the relatively limited number of test samples. The evidence of peroneal nerve SCV, tibial nerve MCV, and median nerve MCV was scarce; hence, it cannot be proven that the Wenyang Huoxue method combined with Western medicine can improve the conduction speed of the aforementioned nerves. The remaining evidence is moderate, suggesting that the Wenyang Huoxue method combined with Western medicine can improve nerve conduction to a certain extent, but more advanced literature is needed to support this evidence. Another finding showed that the Wenyang Huoxue method combined with Western medicine was generally safe; also, most study participants found that their adverse reactions were fewer compared to those taking Western medicine alone.

In the theory of TCM, the Wenyang Huoxue method can improve blood circulation and regulate yin and yang. The Wenyang Huoxue method is generally composed of TCM components such as Ligusticum wallichii, cinnamon twig, Asarum, Caulis Spatholobus, Astragalus and Radix Paeoniae Alba. Ligustrazine is the main component of Ligusticum wallichii, and can accelerate the clearance of blood oxygen free radicals and inhibit aldose reductase activity.^[32]^ Asarum can warm the meridian and has anti-inflammatory and local anesthetic effects. Caulis Spatholobus can improve hemodynamics and has an anti-atherosclerosis effect.^[33]^ Radix Paeoniae Alba has an anti-spasmodic and anti-inflammatory effect.^[34]^ Astragalus membranaceus has an antioxidant free radical formation as well as anti-oxidation, anti-inflammatory, and other pharmacological effects.^[35,36]^

There are some limitations in the study because of the poor quality of the provided research methods and the reported data. First, most of these experiments are small-scale and short-term. Due to the small scale and relatively wide 95% CI, the validity of our statistical analysis may not be robust. Thus, the results may require further verification. Second, almost no Chinese medicine articles related to DPN have been published in English medical journals. Hence, foreign evaluation of Chinese medicine is limited, which reduces its external validity. Third, there is considerable quality heterogeneity among patients in the clinical trials. Different criteria and disease severity levels may limit the universality of our findings. In order to correctly evaluate the safety of TCM, it is necessary to fully reveal its distribution, to reveal the results of the evaluators, and to conduct long-term follow-up. In order to verify the results reported in this paper, large-scale, properly designed the Wenyang Huoxue method treatments of DPN in randomized trials are needed.

## 5 Conclusion

The Wenyang Huoxue method combined with Western medicine seems to create a favorable outcome compared to Western medicine alone, and it appears to be well tolerated in patients with DPN. However, the present evidence is insufficient to support the efficacy and security of the Wenyang Huoxue method combined with Western medicine for DPN due to the poor methodological quality and the limited samples in the included studies. Despite certain limitations, our present findings still have potential implications. Large, properly designed, randomized trials of Wenyang Huoxue method combined with Western medicine used for DPN are required to justify the results reported here. Future trials should overcome the limitations of this review.

## Author contributions

Zhiyuan Deng, Min Liu designed the study. Manjia Wang, Yaohua Fan drafted the paper. Ruilin Huang revised it. Yawen Rao and Xurong Mai developed the search strategies. Zhiyuan Deng and Manjia Wang conducted data collection and analyzed independently. All authors have approved the final manuscript.

Data curation: Zhiyuan Deng, Manjia Wang.

Methodology: Yaohua Fan, Xurong Mai.

Resources: Ruilin Huang, Yawen Rao.

**Conceptualization:** Zhiyuan Deng.

**Data curation:** Zhiyuan Deng, Manjia Wang, Min Liu.

**Formal analysis:** Manjia Wang.

**Methodology:** Yaohua Fan, Yawen Rao, Xurong Mai.

**Resources:** Ruilin Huang.

**Software:** Zhiyuan Deng.

**Writing – original draft:** Zhiyuan Deng, Min Liu.

**Writing – review & editing:** Min Liu.
